# Loss of ARF5 impairs recovery after lysosomal damage

**DOI:** 10.3389/fmolb.2025.1699266

**Published:** 2025-11-10

**Authors:** Martyna O. Iwaniec, Christopher J. Bott, James E. Casanova

**Affiliations:** 1 Department of Cell Biology, University of Virginia Health System, Charlottesville, VA, United States; 2 Intercollegiate Faculty of Biotechnology UG and MUG, University of Gdansk, Gdansk, Poland

**Keywords:** ARF, lysosome, repair, OSBP, ORP

## Abstract

Lysosomal dysfunction is a defining feature of aging and neurodegenerative diseases, where lysosomal membrane permeabilization and release of its contents can trigger cellular death pathways. To counteract this, cells rely on lysosomal quality control mechanisms, many of which depend on lipid delivery to repair damaged membranes. However, the regulatory pathways governing this process remain unclear. In this study, we investigated whether canonical ARF GTPases, best known for their roles in Golgi and endosomal vesicular trafficking, are recruited to damaged lysosomes and contribute to their repair. Using LysoIP-based lysosome isolation, super-resolution immunofluorescence imaging, and functional assays in HeLa and HEK293 cells, we found that ARF1, ARF5, and ARF6 localize to lysosomal membranes following L-leucyl-L-leucine methyl ester (LLOME)-induced permeabilization. While loss of ARF6 did not impair recovery, ARF5 depletion resulted in a nearly complete block of lysosomal repair. These findings identify ARF proteins as early responders to lysosomal damage and suggest isoform-specific roles in coordinating the pathways of lysosomal quality control.

## Introduction

Lysosomes are acidic organelles that serve as terminal degradative compartments within the cell (de Duve, 1959). Their lumen contains multiple hydrolytic enzymes that function optimally at low pH. Lysosome membrane permeabilization (LMP), which occurs in response to a variety of pathogenic conditions (Bahr and Bendiske, 2002) as well as lyso-osmotropic drugs (Zhitomirsky et al., 2018), poses a severe threat to cellular homeostasis (F. Wang et al., 2018). To resolve such stress, cells activate multiple repair pathways that act to restore lysosomal integrity and prevent the release of hydrolytic enzymes which can ultimately lead to cell death (Yang and Tan, 2023).

Several distinct lysosomal repair mechanisms have been described, each operating at different stages following membrane damage. Among the earliest responses are stress granules, recently reported to temporarily plug holes in lysosomal membranes (Bussi et al., 2023), the ESCRT-dependent repair machinery (Radulovic et al., 2018), and the lipid transfer protein VPS13C (X. Wang et al., 2025), which are rapidly recruited to sites of LMP. These responses are followed by the recently identified phosphoinositide-initiated membrane tethering and lipid transport (PITT) pathway which facilitates additional non-vesicular lipid transfer to support membrane restoration (Tan and Finkel, 2022). In cases where membrane integrity cannot be reestablished, damaged lysosomes are selectively removed through lysophagy, a form of autophagy that removes dysfunctional lysosomes (Maejima et al., 2013). Together, these findings highlight the highly regulated and even hierarchical nature of lysosomal repair, the dysregulation of which can have severe consequences on cell viability. Nonetheless, the precise molecular mechanisms responsible for the recruitment and coordination of these pathways remain unclear.

Among candidate proteins with potential roles in lysosomal repair are ADP-ribosylation factor (ARF) GTPases. There are six mammalian ARFs (five in humans) which localize to diverse subcellular compartments, including the Golgi apparatus, endosomes, and the plasma membrane (Sztul et al., 2019). Although not previously reported in lysosomal function, ARFs play important roles beyond their canonical function of regulating vesicular trafficking at the Golgi (Kahn et al., 1992; Myers and Casanova, 2008). In their GTP-bound state, ARFs recruit a wide variety of effector proteins. Interestingly, ARF1 has been reported to recruit oxysterol-binding protein (OSBP), a lipid transfer protein belonging to the greater OSBP-related protein (ORP) family (Mesmin et al., 2013). OSBP, together with the related ORP9, ORP10, and ORP11, are essential components of the PITT pathway, where they promote the establishment of membrane contact sites (MCS) and facilitate bidirectional lipid exchange between ER and lysosomes (Tan and Finkel, 2022). However, the specific mechanisms driving their recruitment remain unresolved. Based on these observations, we hypothesized that ARFs are recruited to permeabilized lysosomal membranes, where they may serve as upstream regulators of OSBP/ORP recruitment and later lysosome-ER MCS formation.

Here, we report that ARF1, ARF5, and ARF6 are recruited to lysosomes upon the induction of membrane damage. While our data do not support ARF-dependent recruitment of ORPs to lysosomes, we demonstrate that ARF5, but not ARF6, is critical for lysosomal recovery following damage. These findings establish a previously unappreciated role of ARF5 in promoting cell survival after lysosomal damage and suggest that ARF GTPases may contribute to lysosomal quality control mechanisms independently of ORP recruitment.

## Methods

### Antibodies and chemicals

The antibodies we used were: rabbit antibody to LAMP1 (D2D11) (Cell Signaling Technology, Inc.), Cat No. 9091S, IF (1:400) WB (1:1200); mouse antibody to LAMP1 (1D4B) from Developmental Studies Hybridoma Bank; mouse antibody to GFP (Proteintech) Cat No.:66002-1-Ig WB (1:100,000); mouse antibody to HA (16B12) (Biolegend) WB (1:4000); rabbit antibody to mCherry (Sigma-Aldrich) Cat No.SAB2702295-100UL WB (1:10,000); rabbit antibody to OSBP (Sigma) Cat no. HPA039227 WB (1:1100) IF (1:100); rabbit antibody to ORP9 from Dr. Neale Ridgway, Dalhousie University, IF (1:1000); mouse antibody to Golgin97 (Molecular probes) Cat No. CDF4 A-21270 WB (1:500); rabbit antibody to ARF5 (Novus Biologicals) Cat No. NBP1-31005 WB (1:2500); sheep antibody to TGN46 (Serotec, Oxford United Kingdom); mouse antibody to Gal-3 (B-2) (Santa Cruz Biotechnology, Inc.) Cat No. sc-25279 IF (1:100); IRDye 800CW donkey anti-mouse secondary antibody (Li-COR) 926-32212 WB (1:10,000); IRDye 680RD goat anti-rabbit secondary antibody (Li-COR) 926-68071 WB (1:10,000); Alexa Fluor 488 donkey anti-mouse (ThermoFisher Scientific (Rockford, IL) IF (1:100); Alexa Fluor 488 donkey anti-rabbit (ThermoFisher Scientific (Rockford, IL) IF (1:100); Alexa Fluor 568 donkey anti-mouse (ThermoFisher Scientific (Rockford, IL) IF (1:100); Alexa Fluor 568 donkey anti-rabbit (ThermoFisher Scientific (Rockford, IL) IF (1:100).

The chemicals we used were as follows: phalloidin Acti-stain™ 488 (cytoskeleton.com) Cat #PHDG1; phalloidin Acti-stain™ 670 (cytoskeleton.com) Cat #PHDN1; phalloidin Alexa Fluor™ Plus 405 (Invitrogen) REF: A30104; Leu-Leu methyl ester hydrobromide (LLOME) (Sigma-Aldrich) L7393; Polybrene Infection/Transfection Reagent (Sigma-Aldrich) TR-1003.

### Cell culture

HeLa and HEK293 cells were maintained in Dulbecco’s modified Eagle medium (DMEM) with 10% FBS and 1% Pen–Strep at 37 °C, 5% CO_2_.

### Plasmids and transfections

The plasmids we used were: pLJC5-TMEM192-3xHA (Addgene); pLKO (Addgene); ARF1-GFP, ARF5-GFP, and ARF6-GFP gifted by Dr. Paul Melancon (University of Alberta, Edmonton, AB, Canada); ARF1-HA, ARF5-HA, and ARF6-HA constructs gifted by Dr. Victor Hsu (Brigham and Women’s Hospital, Boston MA).

All transfections for biochemistry were conducted using PolyJet™ (SignaGen Laboratories, Frederick MD) according to the manufacturer’s protocol. HeLa cells used for imaging were transfected with FuGENE® 4K (Promega, Madison WI) according to the manufacturer’s protocol.

### shRNA knockdowns

Lentivirus particles containing empty vector (pLKO) or ARF-specific shRNAs were generated in Lenti-x-293 cells. Target cells were transduced in the presence of polybrene (0.6 μL/mL), and knockdowns were selected by puromycin (0.25 μL/mL media) treatment initiated 48 h post-infection.

### Stable cell line generation

For the LysoIP protocol, we generated two stable HEK293 cell lines expressing TMEM192 mRFP-3xHA or pLKO vector (control). To establish stably expressing cell lines, cells were plated in 10-cm dishes in DMEM with 10% FBS overnight and infected with 500ul of virus containing media. Puromycin selection was initiated the following day to establish stable populations.

### Lysosome immunoprecipitation (LysoIP)

Lyso-IP was performed based on the protocol described in Abu-Remaileh et al. (2017). HEK293 cells stably expressing TMEM192-mRFP-3xHA or pLKO (empty vector control) that reached full confluency were plated in 10-cm dishes. Cells were subjected to either no treatment or to 1 mM LLOME for 3 h prior to processing. For endogenous ARF5 experiments, cells were lysed the following day. For ARF-GFP construct experiments, cells were transfected with the GFP-tagged constructs and lysed 24 h later.

Briefly, untreated or LLOME treated cells were scraped into KPBS with the addition of protease inhibitors (KPBS+) and centrifuged for 5 min at 350 rcf. Cell pellets were resuspended in 500 µL of KPBS+ and gently homogenized with 20 strokes in a glass homogenizer. Homogenized cells were centrifuged for 5 min at 1000 rcf. An aliquot (30 µL) of each supernatant was saved as an input, and the remaining supernatant was used to incubate with KPBS pre-washed HA beads for 15 min. Immunoprecipitates were gently washed in KPBS and eluted in 22 µL 2X SDS PAGE sample buffer.

#### Immunofluorescence microscopy

HeLa cells were plated on fibronectin-coated glass coverslips (40,000 cells/well). The following day, cells were fixed for 12 min in pre-warmed 4% paraformaldehyde (PFA) and rinsed in PBS. Coverslips were transferred to a humidified chamber and permeabilized for 10 min with 0.2% TX-100, 1% BSA in PBS. Cells were then blocked in 5% BSA in PBS for 1 h at room temperature, followed by incubation with primary antibodies in 1% BSA solution for 1 h. After washing three times (5 min each) with PBS, coverslips were incubated with secondary antibodies in 1% BSA solution for 1 h in the dark. Cells were again washed thrice for 5 min in PBS, mounted onto glass slides using ProLong™ Gold Antifade reagent, and left overnight to cure before imaging.

### Confocal microscopy and image analysis

Coverslips were imaged using a Nikon AX-R resonant scanning confocal microscope with ×60 and ×100 oil immersion objectives. For high-resolution imaging, a Nikon Spatial Array Confocal (NSPARC) system was used. All images were denoised using the Denoise-AI module in Nikon NIS-Elements software. NSPARC-acquired images were additionally subjected to blind deconvolution. All acquisition parameters were optimized using NIS-Elements software prior to image collection.

**FIGURE 1:**
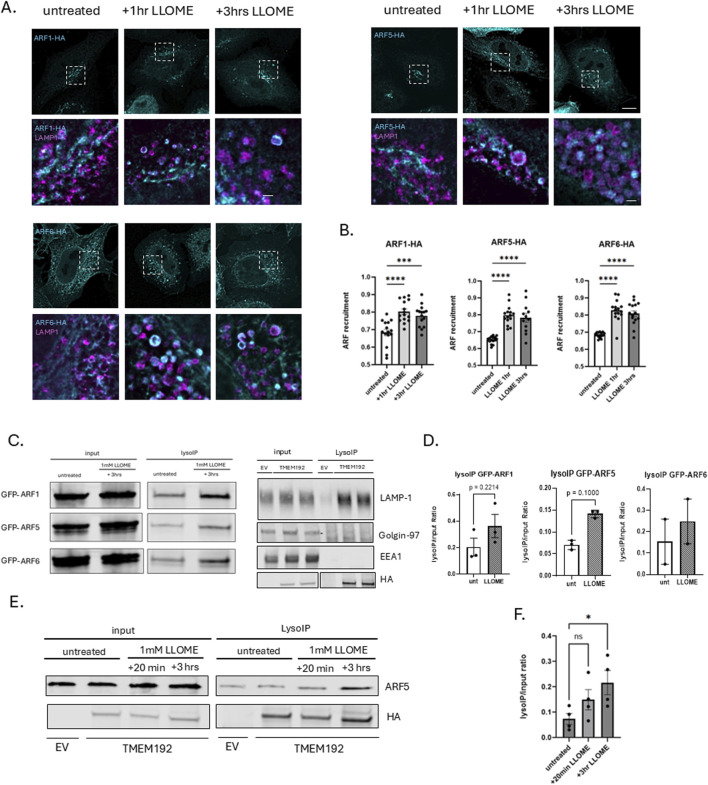
ARF GTPases are recruited to damaged lysosomes. **(A)** HeLa cells were transfected with HA-tagged ARF1, ARF5, or ARF6 and treated the next day with 0.5 mM LLOME for 1 h or 3 h. At each time point, cells were fixed and stained for HA (cyan) and endogenous LAMP1 (magenta) and imaged with super-resolution confocal microscopy (Nikon NSPARC system). Scale bar indicates 10 µm for original size images and 1 µm for zoomed images. **(B)** Quantification of data in **(A)** n = 15 cells. Error bars represent mean +SD. Data were analyzed with one-way ANOVA. *p < 0.05 **(C)** (left) HEK293 cells stably expressing 3XHA-tagged TMEM192 were transfected with GFP-tagged ARF1, ARF5, or ARF6 constructs. Untreated and LLOME-treated (3 h) cells were subjected to lysosome immunoprecipitation using HA-conjugated magnetic beads (LysoIP). Total cell lysates and immunoprecipitates were immunoblotted for GFP to detect bound ARFs (left). Lysates and immunoprecipitates were also immunoblotted for endogenous LAMP1, Golgin-97, or EEA1 (right). **(D)** Quantification of data in **(C)**. Bars indicate mean +SD of ≥2 experimental replicates. Statistical analysis was performed using unpaired t-test. *p < 0.05. Statistics represent comparison of untreated HEK293 cells with 3-h LLOME (1 mM) treated cells quantified by densitometry. **(E)** HEK293 cells were treated with LLOME or not and subjected to the LysoIP protocol. Lysates were immunoblotted and stained for endogenous ARF5 and HA. **(F)** Quantification of data in **(E)**. Bars indicate mean +SD of three experimental replicates. *p < 0.05. Statistics represent comparison of untreated HEK293 cells with 20-min and 3-h LLOME-treated cells quantified by densitometry.

The quantification of ARF recruitment to lysosomes (Figures 1B) was evaluated by the circularity measurement of ARF in untreated and treated conditions. We reasoned that if Golgi has a ribbon-like structure and if lysosomes are circular, then ARFs’ recruitment to damaged lysosomes could be reflected by the increase in ARF circularity in LLOME conditions, compared to the untreated samples. To track that change, we defined an ROI of each cell on the ARF channel. Next, we generated a binary mask of the ARF channel and used a watershed function to separate clustered lysosomes. The measurements were performed for particles ranging from 0.2 to infinity and only for particles above 0.4 circularity. All the above actions were performed in ImageJ (Fiji) software. The circularity measurements obtained for each cell were averaged and included in the statistical analysis.

The quantification of ORP9 recruitment to damaged lysosomes (Figures 3C, D) was performed by first defining a region of interest (ROI) for each cell and generating a binary mask of the 488-nm channel (ORP9). Because lysosomes in untreated cells tend to cluster around the Golgi, which makes it difficult to distinguish lysosomal ORP9 from Golgi-associated ORP9, we applied an intensity-based threshold to exclude the Golgi signal and focus only on untreated peripheral lysosomes. For the lysosomal marker, a binary mask of the 568-nm channel (LAMP1) was created using an identical threshold across all images to ensure consistency. To quantify ORP9 recruitment to LAMP1-positive lysosomes, we calculated the ratio between the mean ORP9 binary intensity overlapping with the LAMP1 binary mask and the mean ORP9 intensity within the ROI.

### Gal3 recovery assay

ARF-depleted HeLa cells were cultured on glass coverslips and treated with 0.5 mM LLOME for 20 min. The treatment medium was then replaced with fresh complete medium, and cells were allowed to recover for either 1 h or 8 h. Following recovery, cells were fixed and immunostained for Galectin-3 (Gal3), LAMP1, and F-actin (phalloidin). Confocal microscopy was used to assess the presence of Gal3-and LAMP1-positive puncta.

### Phalloidin survival assay

HeLa cells transduced with viral vectors were seeded into black-walled 96-well plates (20,000 cells/well). The next day, the cells were either treated or not with LLOME (0.5 mM) overnight or treated with LLOME (0.5 mM) for 20 min followed by recovery in DMEM supplemented with 10% FBS. After overnight incubation, cells were fixed with pre-warmed 4% PFA and washed with PBS. They were permeabilized (0.2% Triton-X-100 in PBS) for 10 min and washed with PBS. Cells were then stained with Alexa-488 phalloidin (1:300) for 90 min and washed three times with PBS. They were imaged using a Cytation 1 imaging microplate reader (BioTek).

### Western blotting

Protein lysates were resolved on 4%–20% SDS-PAGE gradient gels (BioRad, Hercules, CA), then transferred to nitrocellulose membranes using the Trans-Blot® Turbo transfer system (BioRad). Membranes were probed with primary antibodies, followed by fluorescently labeled secondary antibodies, and scanned on a Li-COR Odyssey Clx infrared imaging system. Band intensities (densitometry) were quantified using ImageStudio (LICORbio).

### Statistical analysis

All statistical analyses were performed using GraphPad Prism. Data distribution was assessed with the Shapiro–Wilk normality test, which determined whether parametric or nonparametric tests were applied. Comparisons between two groups were made using unpaired t-tests. For comparisons involving multiple groups, one-way or two-way ANOVA was performed as appropriate, with Dunn’s multiple-comparisons correction. The statistical test used for each figure is specified in the corresponding legend.

## Results

### ARF GTPases are recruited to damaged lysosomes

To investigate whether ARF GTPases are recruited to lysosomes in response to lysosomal membrane permeabilization, we transfected HeLa cells with HA-tagged ARF constructs and exposed them to 0.5 mM L-leucyl-L-leucine methyl ester (LLOME) for various time intervals and imaged via super-resolution (Nikon NSPARC) microscopy (Figure 1). For this purpose, we selected one representative from each of the three ARF classes: ARF1 (class I), ARF5 (class II), and ARF6 (class III). Under untreated conditions, ARF1 and ARF5, as expected, are largely associated with Golgi membranes (Sztul et al., 2019), while ARF6 localizes primarily to the plasma membrane and tubular endosomal compartments but not to lysosomes. However, we observed that ARF1, ARF5, and ARF6 were all recruited to damaged lysosomes (marked by the endogenous lysosomal membrane protein LAMP1) as early as 1 h after the induction of lysosomal membrane damage and remained associated with lysosomes for at least 3 h after treatment (Figure 1A).

To further validate these results, we optimized the previously described Lyso-IP protocol (Abu-Remaileh et al., 2017). Briefly, HEK-293 cells stably expressing a 3XHA-tagged form of the lysosomal membrane protein TMEM192 were transfected with GFP-tagged ARFs and either left untreated or treated with 1 mM LLOME for 3 h. Cells were then homogenized in the absence of detergent and lysosomes precipitated with anti-HA antibodies coupled with magnetic beads. Total cell lysates and precipitated lysosomes were then probed with anti-GFP antibodies to detect bound ARFs (Figure 1B). As shown in Figure 1B, precipitates contained endogenous LAMP1 lacked both the Golgi marker Golgin-97 and early endosome marker EEA1 (D’Souza et al., 2014), demonstrating the purity of the lysosomal preparation. ARF1, ARF5, and ARF6, seemingly present at low levels on precipitated lysosomes, are clarified as being background by the negative control (EV) in the absence of LLOME, but all three ARFs are clearly recruited to lysosomes in response to damage (Figures 1B,C), confirming our imaging results. Focusing on ARF5, we found that the recruitment of endogenous ARF5 begins as early as 20 min after inducing lysosomal damage but is more robust at 3 h post-treatment (Figures 1D,E).

### ARF5 is essential for recovery from lysosomal damage

**FIGURE 2:**
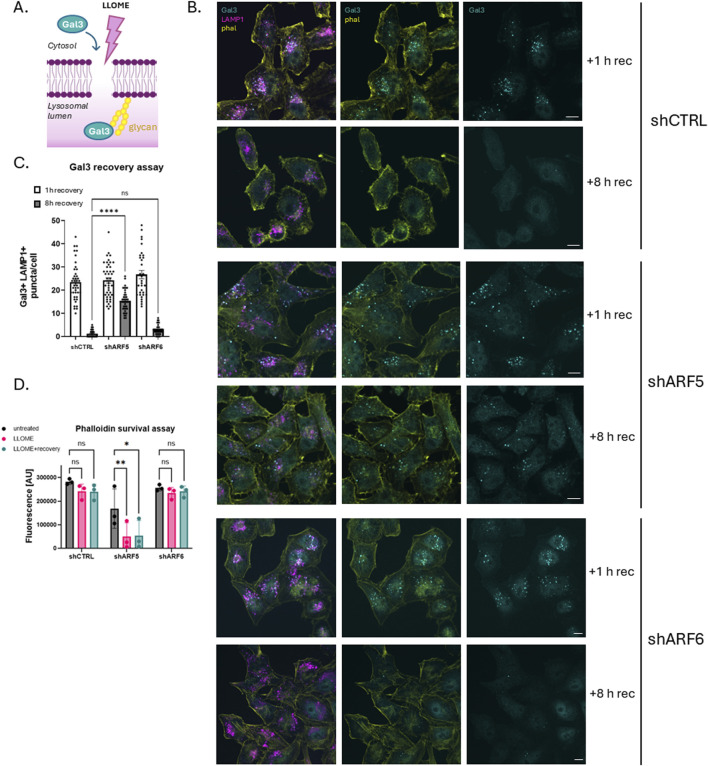
ARF5-depleted cells do not recover from lysosomal damage. **(A)** Schematic of Gal3 recovery assay. **(B)** HeLa cells were transfected with empty vector control (shCTRL), ARF5, or ARF6 shRNAs. Cells were treated or not with LLOME (0.5 mM) which was rinsed out after 20 min of incubation. Cells were allowed to recover for either 1 h or 8 h, after which they were fixed and stained for endogenous Gal3 (cyan), LAMP1 (magenta), and F-actin (yellow) and imaged with confocal microscopy. Scale bar indicates 10 µm. **(C)** Quantification of data in **(B)** n = 40–66 cells from two independent experiments. Error bars represent mean +SD. Data were analyzed with nonparametric Kruskal–Wallis one-way ANOVA test with Dunn’s correction. *p < 0.05 **(D)** HeLa cells were transfected with empty vector (shCTRL), ARF5, or ARF6 shRNAs and plated in black-walled 96-well plates. Cells were then treated with LLOME overnight or after 20 min of incubation, rinsed and allowed to recover (LLOME + recovery). Cells were incubated overnight, fixed the next day, and stained for actin (Alexa-488 phalloidin). Fluorescence was analyzed using a BioTek imaging microplate reader. *y-axis* represents Alexa-488 nm fluorescence [AU]. Data were analyzed with two-way ANOVA with Dunnett’s multiple comparisons test with a single pooled variance.*p < 0.05.

Previous studies have shown that cells can repair damaged lysosomes after washout of LLOME. To determine whether one or more of the recruited ARFs is required for lysosome repair, we monitored the distribution of the cytosolic lectin Galectin-3 (Gal3) by immunofluorescence microscopy. Gal3 is recruited to damaged lysosomes where it recognizes luminal carbohydrates exposed by damage to the limiting membrane (Figure 2A). Upon restoration of membrane integrity, luminal Gal3 is degraded, and cytosolic staining is returned to its diffuse basal state. For this assay, HeLa cells were transduced with lentiviral particles containing either empty viral vector pLKO (shCTRL) or pLKO containing shRNAs targeting ARF5 or ARF6. Cells were then selected with puromycin to ensure a homogenously depleted population. They were treated with LLOME to induce lysosomal damage, washed, and allowed to recover for either 1 h or 8 h (Figure 2B). In control (empty vector) cells, Gal3 puncta were cleared after 8 h, indicating successful lysosomal repair. A similar recovery was seen in ARF6-depleted cells (Figure 2B). In contrast, ARF5 depletion resulted in persistent Gal3 puncta even after 8 h of recovery, indicating a failure to repair lysosomal membranes (Figures 2B,C).

To further validate this observation, we performed a cell survival assay in ARF-depleted HeLa cells (Figure 2D). For this purpose, cells were depleted of ARF5 or ARF6 using shRNA, with empty shRNA vector (shCTRL) as a control. As above, cells were treated with LLOME, washed and allowed to recover for 8 h. Cell survival was monitored by staining with phalloidin and quantified using a fluorescence microplate reader. Consistent with our Gal3 data, ARF5 knockdown led to reduced cell survival following LLOME treatment, while ARF6 depletion had no effect (Figure 2D). Together, these results suggest that ARF5 has an important role in lysosomal membrane repair.

### Lipid transfer proteins OSBP and ORP9 are recruited to damaged lysosomes in an ARF-independent manner

**FIGURE 3:**
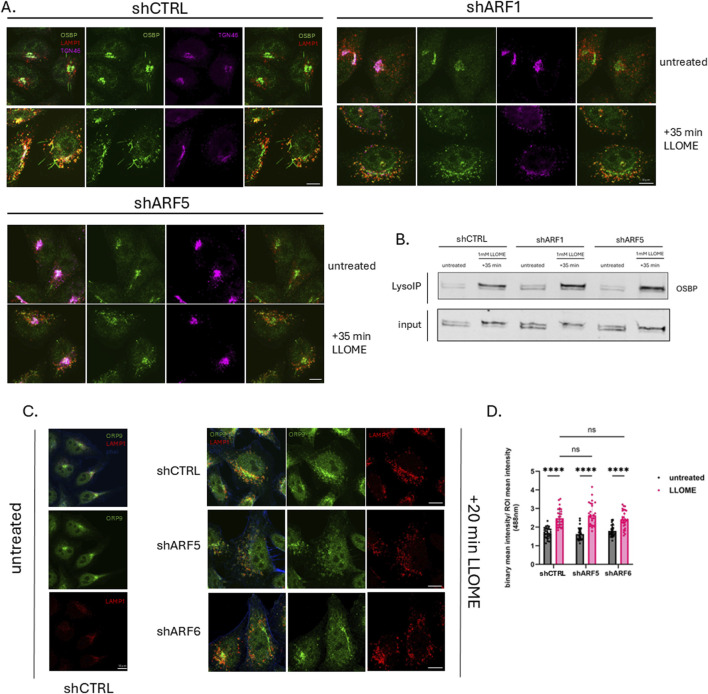
Lipid transfer proteins OSBP and ORP9 are recruited to damaged lysosomes in an ARF-independent manner. **(A)** HeLa cells were transfected with empty vector (shCTRL) or pLKO containing ARF1 or ARF5 shRNAs. Cells were treated or not with LLOME (0.5 mM) for 35 min, fixed and stained for endogenous OSBP (green), LAMP1 (red), and TGN46 (magenta), and imaged with confocal microscopy. Scale bars indicate 10 µm. **(B)** HEK293 cells stably expressing tagged TMEM192 were transfected with empty pLKO vector (shCTRL) or pLKO containing ARF1 or ARF5 shRNAs. Untreated and LLOME (1 mM)-treated cells were subjected to the LysoIP protocol. Precipitated lysosomes were immunoblotted for endogenous OSBP. **(C)** HeLa cells were transfected with empty vector (shCTRL), ARF5, or ARF6 shRNAs. Cells were treated or not with LLOME (0.5 mM) for 20 min, fixed and stained for ORP9 (green), LAMP1 (red), and F-actin (blue), and imaged with confocal microscopy. Scale bars indicate 10 µm. **(D)** Quantification of data in **(C)** n = 28 cells. Error bars represent mean +SD. Data were analyzed with two-way ANOVA with Dunnett’s multiple comparisons test with a single pooled variance. *p < 0.05.

Recent studies have indicated an important role for lipid transfer proteins of the OSBP-related protein (ORP) family in lysosomal membrane repair. Three of these proteins—ORP9, ORP10 and ORP11—are recruited together to lysosomes in the early stages of repair (10–30 min) where they deliver phosphatidylserine (PS) from the ER to lysosomes in exchange for phosphatidylinositol-4-phosphate (PI4P). OSBP, which transfers cholesterol in exchange for PI4P, is recruited slightly later (1 h after induction of damage) (Tan and Finkel, 2022). Previous research demonstrated that under homeostatic conditions, OSBP recruitment to the Trans Golgi Network (TGN) is dependent upon the interaction of both ARF1 and PI4P with the PH domain of OSBP (Mesmin et al., 2013). Based on our findings, we hypothesized that ARF1 or ARF5 may recruit OSBP and/or ORP9/10/11 (the recruitment of which is also dependent on PI4P) to damaged lysosomal membranes to promote ER–lysosome membrane contact site formation. To test this, we first performed imaging of ARF-depleted HeLa cells and found that depletion of ARF1 or ARF5 did not reduce OSBP recruitment to damaged lysosomes (Figure 3A). This was further supported by Lyso-IP analysis in HEK293 cells transduced with viral vectors containing shRNAs targeting ARF1 or ARF5, which similarly showed that loss of either protein did not impair OSBP association with lysosomal membranes after induced damage (Figure 3B).

We next asked whether ARF depletion affects the recruitment of other OSBP-related proteins involved in lysosomal membrane repair. Using the same knockdown approach, we monitored ORP9 localization (as a representative of the PITT complex) by imaging and found that its recruitment to lysosomes was also unaffected by depletion of either ARF5 or ARF6 (Figure 3C). Together, these data suggest that the recruitment of OSBP and ORP9 to damaged lysosomes occurs independently of ARFs.

## Discussion

**FIGURE 4:**
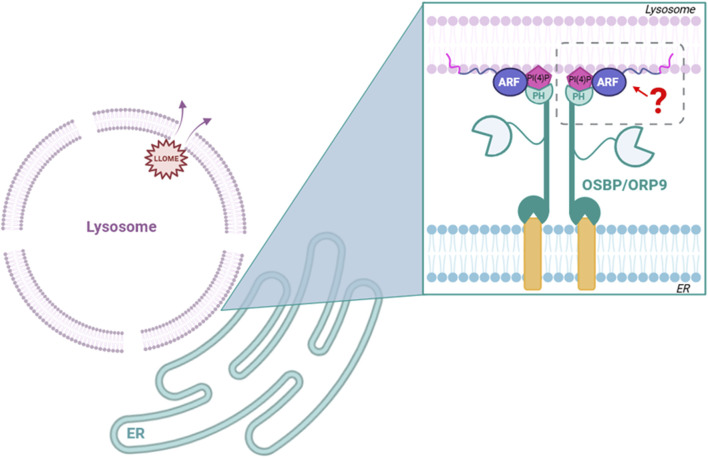
Hypothesis tested: do ARFs mediate interaction of ORP family members with damaged lysosomes? We found that although ARF1, ARF5, and ARF6 are all recruited to damaged lysosomes, none are necessary for the recruitment of ORP family members to these sites.

Lysosomal quality control involves multiple tightly regulated cellular pathways (Yang and Tan, 2023). However, many questions remain unanswered, including how these pathways are coordinated and which signals recruit the necessary repair machinery (Figure 4). In this study, we report that multiple ARF family GTPases are recruited to lysosomes following lysosomal membrane permeabilization. Specifically, we found that ARF1, ARF5, and ARF6 are actively recruited to damaged lysosomal membranes to varying degrees. We selected these three ARFs to represent each of the three ARF classes. However, it is entirely possible that ARF3 and/or ARF4 are also recruited under the same conditions. Functional analyses revealed that among the three ARFs tested, only ARF5 seems to play a significant role in maintaining lysosomal membrane integrity and promoting cell survival after lysosomal stress. This specificity is surprising, as ARF family GTPases share 65% sequence identity and exhibit significant redundancy in many cellular processes (Sztul et al., 2019). ARF5 localizes primarily to the Golgi and ERGIC under homeostatic conditions (Figure Fig1A; Wong-Dilworth et al., 2023), although we recently reported that it also has a role at the plasma membrane in cell migration and adhesion dynamics (D’Souza et al., 2020).

Our preliminary data indicate that ARF5 may be recruited to lysosomes as early as 20 min after damage, suggesting a potential role in early repair processes. With that in mind, we noticed that its recruitment seems to peak at approximately 1 h post LLOME treatment and persists for up to 3 h. We hypothesize that ARF GTPases could be important players in the PITT pathway (Tan and Finkel, 2022) and work as an anchor to recruit OSBP-related proteins to ER/lysosome membrane contact sites. Although ARF1 is known to recruit OSBP to ER/TGN contact sites (Mesmin et al., 2013), we found that neither ARF1 nor ARF5 is necessary for the recruitment of OSBP to damaged lysosomes. We also show that ORP9 (a representative component of the ORP9/10/11 complex), also known to be important in the PITT pathway, does not require ARF5 for its recruitment to damaged lysosomes. It should be noted that we cannot exclude the possibility of functional redundancies among the ARFs. Investigating this would require the simultaneous depletion of multiple ARFs, a process that may have other adverse effects on cellular homeostasis. Nevertheless, ARF5 depletion alone leads to sustained lysosomal damage (based on Gal3 recruitment) and reduces the survival of HeLa cells. This suggests the presence of a previously unrecognized ARF5-dependent lysosomal repair pathway that is critical for maintaining cell viability under lysosomal stress. The observed accumulation of ARF5 at damaged lysosomes 3 hours after LLOME treatment points to a potential role in lysophagy. Interestingly, Rong et al. (2012) implicated ARF1 in lysosomal reformation (ALR), raising the possibility that ARF5 may also participate in this mechanism. Defining the specific role of ARF5 in these processes will require further study.

ARF1 and ARF6 are also recruited to lysosomes in response to damage. However, ARF6 depletion suggested that it either has a redundant role in lysosomal repair pathways or that it is not critical for repair. Thus, the specific functions of ARF1 and ARF6 in this context also remain to be uncovered. Recent reports indicate that the AP-3 adaptor complex, which directly interacts with ARFs, is recruited to lysosomes to facilitate the retrieval of the lipid scramblase ATG9A from lysosomes back to the Golgi (De Tito et al., 2024). Another mechanism contributing to lysosomal quality control is autophagic lysosomal reformation (ALR), which restores the lysosomal pool following the removal of faulty lysosomes through autophagy. ARF1 has been identified as a key regulator of lysosomal tubule fission under serum starvation (Boutry et al., 2023), which might be related to the population of ARF1 we observed on LAMP1-positive lysosomal membranes after 3 h of LLOME treatment. These reports suggest that ARFs can be important players in general lysosomal quality control, but their specific roles on those organelles need further investigation.

It is possible that distinct ARFs coordinate different aspects of lysosomal quality control, such as initiating membrane repair, mediating signaling cascades, exosome/ILV formation, or facilitating contact with other organelles. Alternatively, their recruitment may represent a broader stress response unrelated to the direct repair of lysosomes. Dissecting the molecular functions of individual ARFs in this context will be critical to fully understanding how cells preserve lysosomal integrity. Future studies defining the effectors and pathways engaged by ARFs at damaged lysosomes will provide important insights into the mechanisms that help maintain cellular homeostasis.

## Data Availability

The original contributions presented in the study are included in the article/supplementary material, further inquiries can be directed to the corresponding author.
